# Assessment of phospholipid synthesis related biomarkers for perinatal asphyxia: a piglet study

**DOI:** 10.1038/srep40315

**Published:** 2017-01-10

**Authors:** Ángel Sánchez-Illana, Rønnaug Solberg, Isabel Lliso, Leonid Pankratov, Guillermo Quintás, Ola Didrik Saugstad, Máximo Vento, Julia Kuligowski

**Affiliations:** 1 Neonatal Research Group, Health Research Institute Hospital La Fe, Avenida Fernando Abril Martorell 106, 46026 Valencia, Spain; 2 Department of Pediatric Research, Institute for Surgical Research, University of Oslo, Oslo University Hospital - Rikshospitalet, Sognsvannsveien 20, 0372 Oslo, Norway; 3 Pediatric Department, Vestfold Hospital Trust, Halfdan Wilhelmsens allé 17, 3103 Tønsberg, Norway; 4 Human & Environmental Health & Safety (HEHS), Leitat Technological Center, Avenida Fernando Abril Martorell 106, 46026 Valencia, Spain; 5 Unidad Analítica, Health Research Institute La Fe, Avenida Fernando Abril Martorell 106, 46026 Valencia, Spain; 6 Division of Neonatology, University & Polytechnic Hospital La Fe, Avenida Fernando Abril Martorell 106, 46026 Valencia, Spain

## Abstract

The prompt and reliable identification of infants at risk of hypoxic-ischemic encephalopathy secondary to perinatal asphyxia in the first critical hours is important for clinical decision-making and yet still remains a challenge. This work strives for the evaluation of a panel of metabolic biomarkers that have been associated with the hypoxic-ischemic insult in the perinatal period. Plasma and urine samples from a consolidated newborn piglet model of hypoxia and withdrawn before and at different time points after a hypoxic insult were analyzed and compared to a control group. Time-dependent metabolic biomarker profiles were studied and observed patterns were similar to those of lactate levels, which are currently considered the gold standard for assessing hypoxia. Class prediction performance could be improved by the use of a combination of the whole panel of determined metabolites in plasma as compared to lactate values. Using a multivariate model including lactate together with the studied metabolic biomarkers allowed to improve the prediction performance of duration of hypoxia time, which correlates with the degree of brain damage. The present study evidences the usefulness of choline and related metabolites for improving the early assessment of the severity of the hypoxic insult.

Both in the late preterm and term neonate, hypoxic-ischemic encephalopathy (HIE) secondary to perinatal asphyxia is a leading cause of mortality and acquired long-term neurologic co-morbidities. The overall incidence varies notably: while in developed countries between 1 and 8 per 1000 live births are affected, in low income areas it may account for 26 per 1000 live births^1^.

Perinatal asphyxia is defined as the interruption of blood flow or blood gas exchange to and from the fetus in the perinatal period^2^. Hypoxic-ischemic injury is characterized by its evolution over time. The primary phase (i.e. the hypoxic insult) is followed by a partial recovery during reperfusion; however, in moderate to severe HIE a temporal sequence of injury is set in motion in the latent phase (from ~1–6 h) and the subsequent secondary phase (from ~6 h to >3 days)^3^,^4^.

Perceived prognosis greatly affects clinical management. The most successful intervention for the treatment of moderate to severe HIE is moderate whole body hypothermia. However, treatment has to be initiated within 6 hours from birth^1^. Yet, as the clinical severity of HIE varies over time after the insult, assessments used for diagnosis are time-dependent and their accuracy may be reduced the earlier they are performed^5^. The prompt identification of infants who are most at risk of developing moderate to severe HIE in the critical first hours is desirable as it would help to guide clinical decision making and/or establish a prognosis. Yet, this still remains a challenge.

To date, the diagnosis of an asphyctic process that evolves to HIE is based on prenatal clinical information (sentinel events), and postnatal evaluation using serial Apgar score determinations with special emphasis on neurological assessment and cord blood gas analysis reflecting metabolic acidosis and increased lactate concentration^6^. Amplitude-integrated electroencephalography (EEG), brain magnetic resonance imaging (MRI) and multichannel EEG later on further confirm the diagnosis and the degree of severity^3^,^5^. A number of biochemical markers such as proteins apparently specific for neuronal tissue (creatine kinase brain band, protein S100B, neuron-specific enolase) and proteins involved in the pathogenesis of traumatic brain injury (e.g. glial fibrillary acidic protein, ubiquitin carboxyl-terminal hydrolase L1, phosphorylated axonal neurofilament heavy chain) as well as circulating pro-inflammatory cytokines (interleukin 1β and 6) and circulating mRNAs, among others, have been studied^3^,^5^,^7^,^8^,^9^. In most cases their usefulness has only been shown in pilot studies and currently none has entered into routine clinical use^9^,^10^. Furthermore, issues about the specificity of the reported markers have been raised^9^ and information on correlation with long time outcomes is lacking^5^.

Animal studies seeking after novel biomarkers which are able to provide improved performance have been carried out^11^. In a previous targeted metabolomic study in newborn piglets plasma it was shown that the duration and intensity of hypoxia were more accurately reflected by ratios of alanine to branched-chained amino acids (BCAA) and glycine to BCAA than by the traditionally employed plasma lactate concentration^12^. With the aim of discovering early biomarkers Solberg *et al*.^13^ carried out an untargeted metabolomics study involving the analysis of retinal tissue samples from a piglet model of perinatal asphyxia. Retina is an integral neural tissue with a high metabolic demand for oxygen supported by an efficient vascular supply in which, under hypoxic conditions, a series of adaptive responses are induced including changes in the blood flow, angiogenesis, and protective metabolic adaptations^14^. After the hypoxic insult, elevated levels of CDP-choline, the limiting intermediate compound in the major pathway of phosphatidyl-choline biosynthesis (i.e. the Kennedy pathway)^15^ were found with concentrations correlating with the intensity of retinal hypoxia. In parallel, *in vivo* mouse^16^ and rat models^17^, revealed increased choline levels in brain tissue after hypoxia-ischemia in comparison to sham-controls. Earlier, in an *ex-vivo* rat model a decrease of choline in brain tissues after oxygen-glucose deprivation has been reported comparing hypothermia and normothermia groups^18^.

Based on these findings in neuronal tissue, studies in minimal-invasively obtained biofluids were carried out. Choline and cytidine, two of the precursors of CDP-choline, were found among a set of 21 metabolites showing significant changes in a liquid chromatography-time-of-flight-mass spectrometry (LC-TOF-MS) untargeted metabolomics study on plasma samples from piglets subjected to hypoxia and reoxygenation in comparison to a non-asphyxiated control group^19^. Skappak *et al*.^20^ found elevate levels of betaine, which is metabolically related to choline, in urine samples obtained from asphyxiated piglets vs. non-asphyxiated controls after 6 h of hypoxic insult. In concordance with the discussed results, a study involving the analysis of umbilical cord serum from newborns revealed an increase in choline and/or betaine levels in conditions of asphyxia and HIE^21^.

Based on the above-cited observations a target study of three precursors of CDP-choline (choline, cytidine and uridine), together with betaine was incentivized. This work strives for the validation of candidate biomarkers in plasma and urine that have been associated with the hypoxic-ischemic insult in the perinatal period as they could potentially be of importance for grading the intensity and duration of tissue hypoxia in the clinical setting helping to stratify patients that could benefit from early moderate therapeutic hypothermia.

## Results

### Characterization of the study cohorts

**Figure 1:**
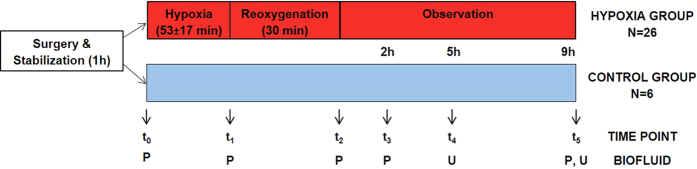
Overview of the study design. Note: P stands for plasma, U stands for urine; no plasma samples have been collected from animals included in the control group at t_2_.

The experimental study design and sample collection time points are shown in Fig. 1. Table 1 summarizes parameters and variables continuously monitored during the animal experiments including hemoglobin, base excess (BE), mean arterial blood pressure (MABP), partial O_2_ arterial pressure (pO_2_) and partial arterial CO_2_ pressure (pCO_2_). No differences between control and hypoxia groups were found neither for the basic biologic characteristics nor the clinical parameters after 1 h of stabilization. However, at the end of hypoxia in the intervention group significantly lower pH, BE, and MABP levels were found, while no difference in heart rate was observed (see Table 1). Resuscitation with room air rapidly improved clinical variables in the intervention group and at the end of resuscitation both groups showed comparable levels of pO_2_, pCO_2_ and MABP.

### Effect of hypoxia on plasma and urine samples

**Figure 2:**
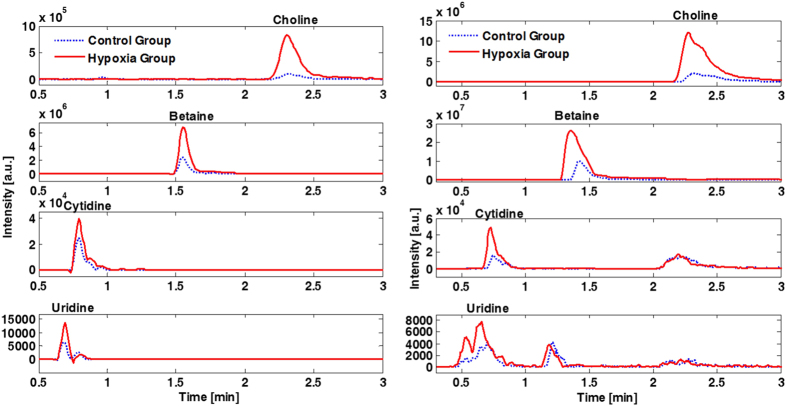
Representative chromatograms of plasma (left) and urine (right) samples from control and intervention (hypoxia) groups.

**Figure 3:**
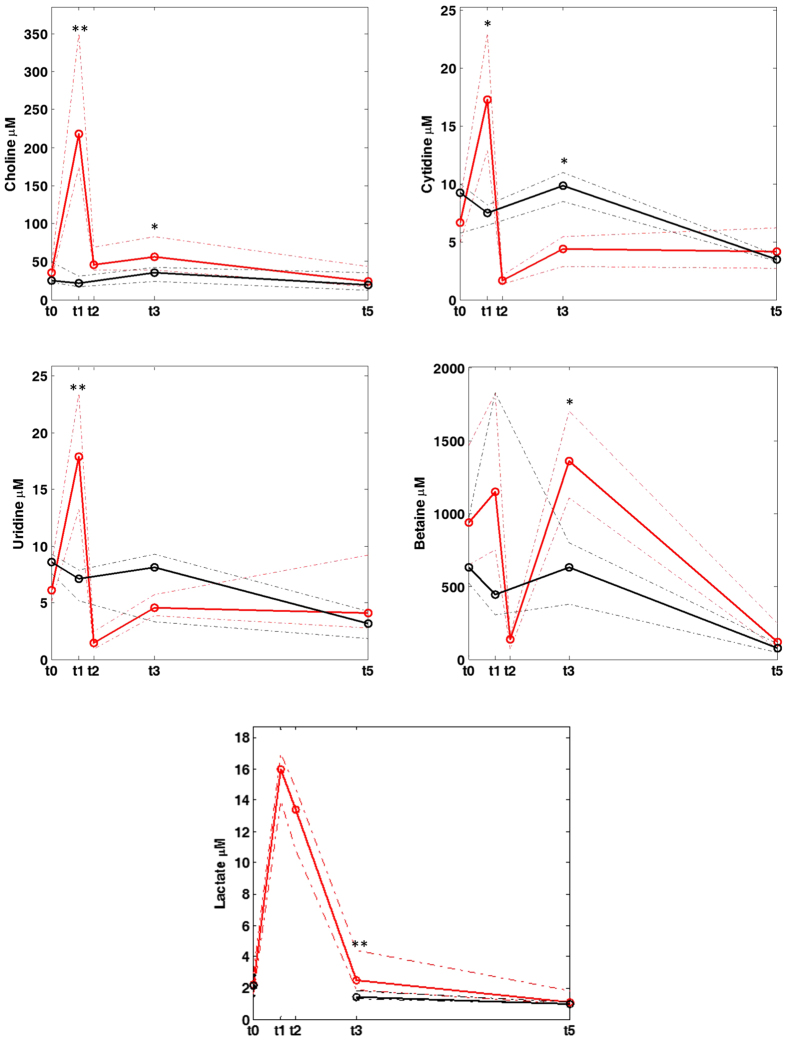
Concentrations of metabolites in plasma samples at different studied time points. Note: * and ** indicate significant differences (p-value < 0.05 and 0.01, respectively) between samples from the control (black line) and intervention groups (red line).

**Figure 4:**
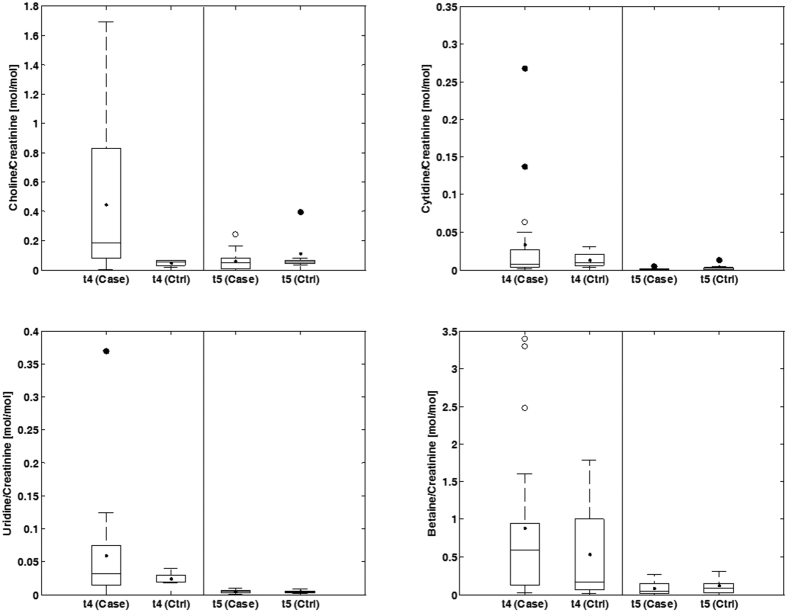
Boxplots representing concentrations of metabolites in urine. Note: Case = intervention group; Ctrl = control group; concentrations were normalized by creatinine.

Choline, betaine, cytidine and uridine were determined in plasma and urine samples employing ultra performance LC coupled to tandem MS (UPLC-MS/MS). Representative chromatograms of samples from the control and hypoxia group obtained directly after asphyxia (t_1_) are shown in Fig. 2. Concentrations of the studied metabolites in plasma and urine samples as determined employing the UPLC-MS/MS method at the different time points are represented in Figs 3 and 4, respectively. In addition, the plasma lactate profile at the same time points is shown in Fig. 3 for the sake of comparison. In plasma no significant changes in concentrations were detected in the control group with the exception of a decrease in betaine and cytidine between 2 and 9 h after reoxygenation (i.e. t_3_ and t_5_, respectively). In contrast, a highly significant, abrupt rise was observed in the intervention group for choline, cytidine and uridine levels when comparing plasmatic concentrations before and at the end of hypoxia (i.e. at t_0_ and t_1_, respectively) followed by a descent when comparing levels at the end of hypoxia to those found 2 h and 9 h after reoxygenation (i.e. t_3_ and t_5_, respectively). Consequently, at the end of hypoxia the intervention group showed significantly increased concentrations of choline, cytidine and uridine (Wilcoxon rank sum, *p*-value < 0.05) as compared to the control group. This difference remained significant (Wilcoxon rank sum, *p*-value < 0.05) for choline even after 2 h of reoxygenation (t_3_). The profile of those three metabolites is similar to changes observed in lactate levels. For betaine a slightly different profile was obtained: the increase of betaine in the intervention group at t_1_ was not found to be significant; this was followed by a decrease at t_2_ and then an increase at t_3_ yielding significantly higher concentrations (Wilcoxon rank sum, *p*-value < 0.05) in the intervention group. Besides, a significant decrease in the plasmatic concentrations was observed for both control and hypoxia groups in samples withdrawn 9 hours after reoxygenation (t_5_). In urine samples no statistically significant changes were observed. However, choline concentrations in the intervention group showed a trend to higher concentrations and higher between-individuals variability.

### Prognostic capacity of the studied metabolites

With the aim of assessing the prognostic capacity of the studied biomarkers, receiver operating characteristics (ROC) curves and areas under the ROC curve (AUC) were calculated for lactate, choline, cytidine, uridine and betaine comparing control vs intervention groups at each studied time point in urine and plasma samples^22^. The obtained AUC values and their 95% confidence interval (CI) are listed in Table 2. In plasma collected before initiating hypoxia (t_0_), no statistically significant models were obtained. Directly after hypoxic insult (t_1_) choline, cytidine and uridine showed AUC values ≥0.969. As anticipated from the concentration profiles discussed above, the prognostic power was smaller 2 h after reoxygenation (t_3_); however, for choline and cytidine, as well as lactate AUC of ≥0.814 were obtained. The effect of hypoxia on the studied metabolites 9 h after reoxygenation (t_5_) was negligible and none of the calculated AUC values showed a better prediction performance than random models in both studied biofluids. In addition, choline concentrations determined in urine samples at t_4_ showed a statistically significant prediction power.

**Figure 5:**
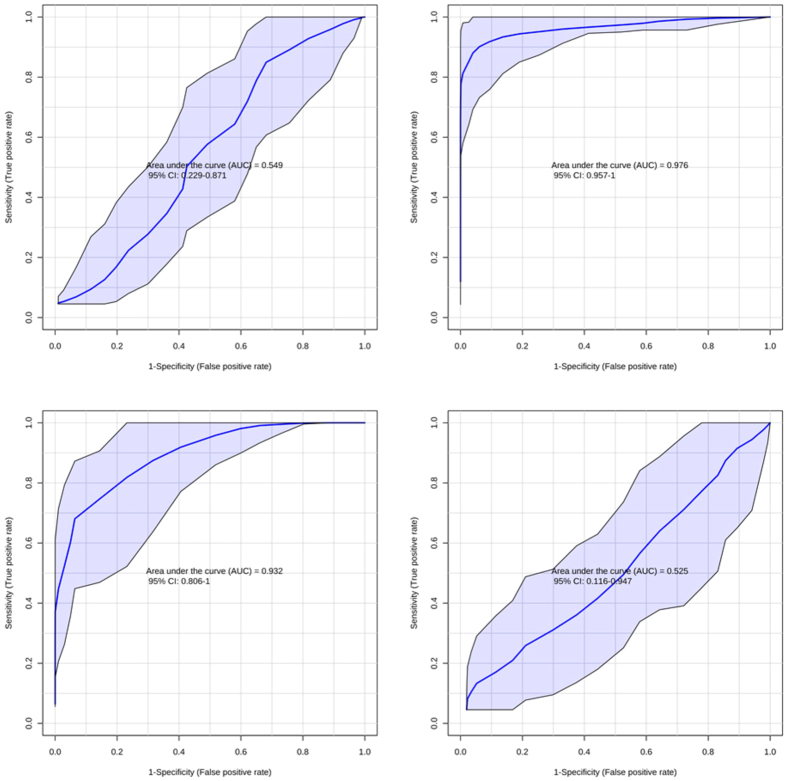
Multivariate ROC curves comparing cases and controls at t_0_ (left, top), t_1_ (right, top), t_3_ (left, bottom) and t_5_ (right, bottom).

Additionally to univariate ROC curves, multivariate ROC curves were calculated for each time point and biofluid using data from all available metabolites, thereby yielding optimum prediction properties. AUC (95% CI) values are shown in Table 2. Statistically significant models were obtained directly after asphyxia (t_1_) and 2 h after reoxygenation (t_3_). It is noteworthy that the predictive power 2 h after reoxygenation could be improved by the multivariate approach. Figure 5 shows ROC curves of multivariate models calculated for t_0_, t_1_, t_3_ and t_5_ in plasma samples. This figure illustrates the changing prediction power in dependence of the timing of blood sample collection.

### Correlation with time of hypoxia 2 h after reoxygenation (t_3_)

**Figure 6:**
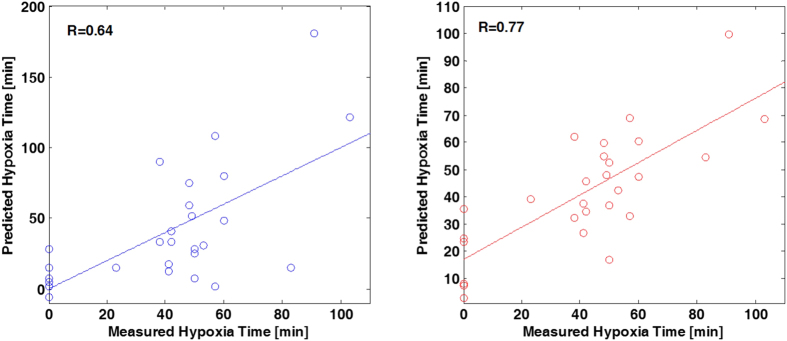
Correlation of lactate levels in blood with the duration of hypoxia (left) and predicted *vs* measured hypoxia time using a PLS multivariate model based on the levels of lactate, choline, cytidine, uridine and betaine (right) at t_3_.

The correlation of lactate levels in blood with the duration of hypoxia has been studied (see Fig. 6, left) obtaining a coefficient of correlation (R) of 0.64, a slope significantly different from zero (*p*-value < 0.01) and a standard deviation of the residuals of ±33 min. This result was compared to the performance of a Partial Least Squares (PLS) model using the plasma levels of lactate with choline, cytidine, uridine and betaine and 1 latent variable (LV) (see Fig. 6, right). For the PLS predicted *vs* measured hypoxia time an R of 0.77 was obtained with a slope significantly different from zero (*p*-value < 0.01) and a standard deviation of the residuals of ±14 min.

## Discussion

Assessing the degree of perinatal asphyxia in the immediate postnatal period still remains a challenge. However, this information would be extremely valuable for optimizing therapy and reliably predicting short-and/or-long term outcomes especially in low-income countries with little access to hypothermia therapy.

An ideal biomarker is one that is easily and rapidly performed, its concentration is proportionately changed in the course of the disease according to the degree of injury and thus it can be used as an early predictor of long-term outcomes. Data presented in Table 1 demonstrate that physiological variables do not provide sufficient predictive capacity as they return to normal values during resuscitation. In this study we assessed and compared the evolution of four metabolites, namely choline, betaine, cytidine and uridine, which have been identified as potential biomarkers of hypoxia in previous studies^13^,^19^,^20^,^21^. Levels of lactate, which are currently considered the gold standard for assessing asphyxia in the clinics, were used throughout this work as reference for comparison.

The present study shows significant transient changes in plasmatic levels of the studied metabolites in a piglet model of hypoxia-reoxygenation (see Fig. 3). Whereas choline, cytidine and uridine followed a similar profile as compared to lactate levels, betaine, showed a slightly different pattern (see Fig. 3). In urine samples collected 5 h after the insult (t_4_), less pronounced differences in choline concentrations were found (see Fig. 4). Choline is involved in a number of physiological processes. Hence, it is converted into betaine in a two-step enzymatic reaction taking place in the mitochondria of liver and kidney where it acts as an osmolyte to control physiologic osmotic pressure^23^,^24^. In the brain, choline together with the pyrimidines cytidine and uridine is incorporated into phosphatidylcholine following the cytidine 5-diphosphocholine pathway discovered by E.P. Kennedy in 1954^25^. Their uptake from the circulation into the brain’s extracellular fluid is carried out by means of nucleoside transporters located at the blood-brain-barrier (BBB). The rate at which uptake occurs constitutes a major factor determining phosphatide synthesis^26^. Hence, these metabolites are precursors for the synthesis of membrane phospholipids including phosphatidylcholine, and thereby affect signaling and transport across membranes^15^,^24^. In newborns, both, the endogenous biosynthesis of phosphatidylcholine and the uptake from mother’s milk have been studied^27^. In addition, choline’s function as a part of the neurotransmitter acetylcholine has been discovered at the beginning of the 20^th^ century^28^,^29^. Previous observations of elevated CDP-choline levels in retinal tissue support the alteration of the Kennedy pathway rather than the formation of acetylcholine as acetylcholine levels in the studied neuronal tissue remained unaffected during hypoxia-reoxygenation^13^.

The evolution of the plasmatic profiles of these metabolites could potentially be related with the alteration of the Kennedy pathway together with the disturbance of the function of the BBB which has been reported during HI insults in neonates^30^. In addition, a recent study on neonatal mouse brain has revealed the transient opening of the BBB within early hours after the insult^31^. Future studies will focus on the elucidation of the mechanism behind the reported observations.

Poor predictive capacity was observed before hypoxia (t_0_) and 9 h after reoxygenation (t_5_), whereas directly after asphyxia (t_1_) and 2 h after reoxygenation (t_3_) (in plasma) and at 5 h after reoxygenation (t_4_) (in urine) significant models for the prediction of hypoxia were obtained (see Table 2 and Fig. 5). In view of the clinical applicability as valuable biomarkers of these metabolites, the time-course is of great importance. In the clinical setting, there is no access to blood samples before hypoxic insult for the sake of a relative comparison of metabolic changes. Hence, in this study all ROC curves were constructed comparing control and intervention groups at each time point. Furthermore, the selection of the most appropriate therapeutic strategy is limited by the therapeutic window of 6 h from birth for hypothermia treatment. At present, the gold standard of metabolic biomarkers for assessing the severity of hypoxia is lactate. In this study, lactate showed a good predictive power in plasma collected 2 h after reoxygenation (t_3_) (see Table 2). However, it is noteworthy that the performance could be improved (AUC from 0.898 to 0.932 and CI reduced by >20%) by the use of a combination of the whole panel of determined metabolites. An interesting finding from the viewpoint of a potential clinical application of these biomarkers is that the perturbation persists during at least a couple of hours in plasma and even longer in urine. This offers the possibility to carry out serial determinations within the first hours of life, which could help to guide clinical decisions on treatment providing complementary information to other available diagnostic tools in the delivery room.

Another interesting finding of the present study is the prediction performance of choline in urine samples collected 5 h after reoxygenation (t_4_) which to date, to the best of our knowledge, has not yet been reported in scientific literature. This finding is of special interest for the clinics, due to the non-invasive character of urine samples. However, metabolic fluctuations in urine reflect a much longer time span as compared to plasma samples and therefore, their interpretation and significance in the context of an acute process is more complex.

The duration of hypoxia is known to be directly proportional to the degree of brain damage^32^. Hence, the correlation of the studied biomarkers with the measured time of duration of hypoxia was assessed. By combining choline and its related metabolites with lactate levels measured 2 h after reoxygenation (t_3_), the coefficient of correlation could be improved by >20% from 0.64 to 0.77 as proven in Fig. 6. Furthermore, the standard deviation of the residuals was reduced from 33 to 14 min thereby improving the prediction precision by 58%. This corroborates the usefulness of the described biomarkers for the clinical diagnosis within the first hours of life.

To summarize, the present study showed the potential of choline and related metabolites as biomarkers for hypoxia. The selected panel of metabolites was able to improve the predictive performance of lactate and further helped to improve the prediction precision of the duration of hypoxia. Their applicability for clinical diagnosis is to be confirmed in multicenter trials involving the analysis of blood and urine samples from newborns suffering from HIE. These studies will also focus on the assessment of their correlation with long-term neurodevelopmental outcomes.

## Methods

### Ethics statement

Animal experiments were carried out at Oslo University Hospital (Norway) with the study protocol being approved by the Norwegian Council for Animal Research (approval number 3399). Researchers certified by the Federation of European Laboratory Animals Science Association (FELASA) cared for and handled the animals in accordance with the European Guidelines for Use of Experimental Animals.

### Animal model

32 newborn Noroc (LyxLD) pigs aged between 12 h and 36 h, with hemoglobin (Hb) levels >5 g dL^−1^ and good general conditions were included in the study. Anesthesia was induced with sevofluran 5%, then an ear vein was cannulated, sevofluran was disconnected and the piglets were given pentobarbital sodium 20 mg kg^−1^ and fentanyl 50 mg kg^−1^ intra venous (IV) as bolus injections. Continuous infusion of fentanyl (50 μg kg^−1^ h^−1^) and midazolam (0.25 mg kg^−1^ h^−1^) was employed to maintain anesthesia. The piglets were orally intubated, ventilated and surgically prepared as described by Andresen *et al*.^33^. At the end of the observation time, the animals were given an overdose of pentobarbital (150 mg kg^−1^ h^−1^ IV).

After 1 h of stabilization, the piglets were randomly assigned either to the hypoxia and reoxygenation group (intervention group, n = 26) or the control group (n = 6) without exposure to hypoxia, but maintaining the same procedures and observation times (anesthesia, surgery, ventilation and sample collection). In the intervention group, hypoxemia and subsequently hypoxia-ischemia was achieved by ventilation with a gas mixture of 8% O_2_ in N_2_ until either the mean arterial blood pressure (MABP) decreased to <20 mm Hg or the base excess (BE) reached −20 mM L^−1^. CO_2_ was added during hypoxemia aiming at a pCO_2_ of 8.0–9.5 kPa (60–71.3 mmHg) in order to imitate perinatal asphyxia. After 30 min of reoxygenation employing room air (21% O_2_, n = 12) or 2.1% H_2_ gas mixed into synthetic air (n = 14) all animals were kept normocapnic with pCO_2_ between 4.5 and 5.5 kPa (33.8–41.3 mmHg) during 9 hours receiving room air. Continuous surveillance of blood pressure, saturation, pulse, temperature, and blood gas measurements were performed. In this study both reoxygenation groups were merged together (intervention group, n = 26) as no statistically significant differences could be found between animals from both groups in a previous untarget metabolomics study^19^.

Whole blood samples from piglets included in the hypoxia group were taken in ethylene-diamine-tetraacetic acid (EDTA) Vacutainer® blood collection tubes before start of hypoxia (t_0_), at the end of hypoxia (t_1_), after rexoygenation (t_2_) and 2 and 9 hours after reoxygenation (t_3_ and t_5_, respectively). Blood volumes drawn for testing were replaced by 1.5× of saline. Plasma was obtained immediately after sampling by centrifugation of whole blood samples at 2000 × g for 10 min at 4 °C. For those piglets included in the control group plasma were also collected after 1 h stabilization. Besides, control plasma samples were collected at time points matching the mean values of the end of hypoxia (t_1_), t_3_ and t_5_ for the comparison of the metabolic profiles in both groups of samples. Urine samples were withdrawn from piglets included in the hypoxia group 5 and 9 hours after reoxygenation (t_4_ and t_5_). Urine samples from piglets included in the control group were also collected at the same time point for the analysis of the effect of hypoxia and reoxygenation in the urinary metabolic profiles. Plasma and urine samples were stored at −80 °C until analysis. The experimental study design and sample collection time points are visualized in Fig. 1.

### Chemicals and reagents

Solvents of LC-MS grade and were purchased from Scharlau (Barcelona, Spain). Pure analytical standards (choline bitartrate, betaine, cytidine and uridine) and ammonium formate with purities ≥98% were from Sigma-Aldrich Química SA (Madrd, Spain) and betaine-D_11_ (98%) from Cambridge Isotope Laboratories Inc. (Tewksbury, MA, USA).

### Sample preparation

Samples were thawed on ice and homogenized. 190 μl of cold acetonitrile (4 °C) and 10 μl of internal standard solution (betaine-D_11_) at a concentration of 10 μM were added to 10 μl of plasma or urine. Samples were centrifuged at 10000 × *g* for 10 min at 4 °C. 100 μl of supernatant were collected and transferred to a 96 well plate for LC-MS/MS analysis. During sample processing, samples were maintained on ice in order to prevent sample degradation. Blanks were prepared by replacing the sample volume with H_2_O. Quality control (QC) samples for plasma and urine were prepared by mixing 5 μl of each sample. QCs were processed as described for samples.

In urine samples creatinine levels were determined for normalization of biomarker concentrations employing a MicroVue Creatinine Assay Kit (ref. 8009) from Quidel Corporation (San Diego, CA, USA).

### Quantitative ultra-performance liquid chromatography coupled to tandem mass spectrometry (LC-MS/MS) analysis

Quantitative analysis of choline, betaine, cytidine and uridine was performed employing an Acquity UPLC system coupled to a Xevo-TQ triple quadrupole MS detector operating in the positive electrospray ionization mode (ESI^+^) (Waters, Manchester, UK). With a total runtime of 5 min, isocratic elution was performed using a Kinetex HILIC column (100 × 2.1 mm, 1.7 μm, 100 Å) from Phenomenex (Torrance, CA, USA) and a 30:70 v/v H_2_O:CH_3_CN mobile phase at pH 7 containing 5 mM ammonium formate. Flow rate, column temperature and injection volume were set at 0.4 ml min^−1^, 30 °C and 5 μl, respectively. Detection conditions were set as follows: capillary voltage to 3.5 kV, source temperature to 120 °C and the cone, desolvation and collision gas flows were 50 L h^−1^, 700 L h^−1^ and 0.2 mL min^−1^, respectively. Dwell time was set to 5 ms ensuring a minimum of 10 data points per peak.

Stock solutions of standards were prepared in water by direct weighing. A set of 12 standard solutions was obtained by serial dilution of the stock solution in mobile phase covering the concentration ranges indicated in Table 3. For quantification, tandem MS detection was carried out by multiple reaction monitoring (MRM) applying the acquisition parameters shown in Table 3. Individual standard solutions at a concentration of 10 μM were used for optimizing ionization and fragmentation parameters as wells as for confirming the absence of spectral interferences between the studied compounds.

An initial system suitability test was carried out at the beginning of each batch involving the analysis of blank samples and solvent blanks to assure appropriate sensitivity levels and reproducible retention times (±0.2 min). QC samples spiked with the stock solution were intercalated in the sample batch measurement to detect deficiencies in accuracy and precision levels prior to the release of results. Accordingly, at least 75% of the values found for the QC standards should be within ±25% of their respective nominal values to accept the batch.

### Data processing

Raw data were acquired and processed using MassLynx 4.1 and QuanLynx 4.1 (Waters, Milford, MA, USA), respectively. Linear response curves were obtained from UPLC-MS/MS peak area measurements employing betaine-D_11_ as internal standard. Further data processing was carried out in Matlab 2015a from Mathworks Inc. (Natick, MA, USA) using the PLS Toolbox 8.0 from Eigenvector Research Inc. (Wenatchee, WA, USA) and in-house written functions. ROCs and AUCs were computed employing MetaboAnalyst 3.0^34^. Missing values were estimated using *k*-nearest neighbors and data were autoscaled. For multivariate ROC curve based exploratory analysis all available features at each time point were employed. Feature ranking was based on univariate AUC values and random forests were used as a classification method. ROC curves were generated by Monte-Carlo cross validation (MCCV) using balanced subsampling where in each MCCV two thirds of the samples were used to evaluate the feature importance. Then, the model was validated using one third of the samples that were left out during model generation. For the calculation of the CI, this procedure was repeated 500 times.

## Additional Information

**How to cite this article**: Sánchez-Illana, Á. *et al*. Assessment of phospholipid synthesis related biomarkers for perinatal asphyxia: a piglet study. *Sci. Rep.* **7**, 40315; doi: 10.1038/srep40315 (2017).

**Publisher's note:** Springer Nature remains neutral with regard to jurisdictional claims in published maps and institutional affiliations.

## Figures and Tables

**Table 1:** Physiological background data. Characterization of the study cohort (intervention group) before (t_0_), directly after asphyxia (t_1_) and after reoxygenation (t_2_–t_5_) and at corresponding time points for the control group.

Parameter/Variable	Control Group	Intervention Group
Weight [g]	1810 (±173)	1889 (±127)
Age [h]	28.7 (±3)	25.6 (±4)
Gender [male/female]	3/3	12/14
Hypoxia Time [min]	0	53.4 (±17)
Hb [g 100 mL^−1^]		
t_0_	7.2 (±1.0)	7.3 (±1.1)
t_5_	6.7 (±0.8)	6.8 (±0.05)
pH		
t_0_	7.41(±0.04)	7.44 (±0.07)
t_1_	7.42 (±0.03)	6.86 (±0.07)
t_2_	7.44 (±0.03)	7.16 (±0.07)
t_3_	7.46 (±0.03)	7.39 (±0.08)
t_4_	7.42 (±0.05)	7.40 (±0.09)
t_5_	7.44 (±0.08)	7.40 (±0.06)
BE [mmol L^−1^]		
t_0_	2.25 (±2.8)	1.89 (±3.4)
t_1_	1.98 (±2.7)	−19.3 (±2.2)
t_2_	1.98 (±2.7)	−14.8 (±2.4)
t_3_	2.33 (±2.6)	−0.4 (±4.2)
t_4_	0.66 (±4.2)	−0.7 (±4.8)
t_5_	−0.01 (±4.7)	−0.3 (±5.2)
MABP [mm Hg]		
t_0_	49.4 (±4.9)	54.4 (±8.1)
t_1_	48.2 (±7.3)	22.7 (±8.1)
t_2_	48.2 (±7.3)	41.9 (±11.0)
t_3_	46.7 (±6.2)	47.5 (±11.3)
t_4_	47.6 (±8.3)	44.2 (±9.3)
t_5_	47.0 (±13)	45.7 (±10.8)
Heart rate [beats min^−1^]		
t_0_	147 (±11)	144 (±26)
t_1_	156 (±20)	160 (±47)
t_2_	156 (±20)	200 (±35)
pO_2_ [kPa]		
t_0_	9.9 (1.0)	10.6 (1.7)
t_1_	10.3 (0.8)	5.0 (0. 6)
t_2_	10.3 (0.8)	10.7 (1.4)
pCO2 [kPa]		
t_0_	5.4 (0.5)	5.2 (0.8)
t_1_	5.1 (0.3)	9.3 (1.1)
t_2_	5.1 (0.3)	4.7 (0.8)

Values are presented as mean (±s). Hb = hemoglobin; BE = base excess, MABP = mean arterial blood pressure; pO_2_ = partial O_2_ pressure; pCO_2_ = partial CO_2_ pressure.

**Table 2:** AUC (CI 95%) for biomarkers for hypoxia comparing control vs. intervention groups.

Metabolite	Plasma at t_0_	Plasma at t_1_	Plasma at t_3_	Plasma at t_5_	Urine at t_4_	Urine at t_5_
Lactate	0.518 (0.306–0.736)	—	0.898 (0.746–0.992)	0.561 (0.301–0.816)	—	—
Choline	0.615 (0.276–0.926)	1 (1–1)	0.814 (0.614–0.955)	0.577 (0.356–0.792)	0.829 (0.612–0.961)	0.630 (0.379–0.815)
Cytidine	0.679 (0.394–0.926)	0.969 (0.892–1)	0.826 (0.568–1)	0.574 (0.393–0.765)	0.592 (0.296–0.823)	0.713 (0.481–0.713)
Uridine	0.641 (0.372–0.878)	1 (0.969–1)	0.629 (0.326–0.909)	0.667 (0.429–0.866)	0.632 (0.414–0.842)	0.500 (0.231–0.787)
Betaine	0.615 (0.308–0.843)	0.708 (0.334–0.973)	0.788 (0.439–0.981)	0.673 (0.413–0.898)	0.671 (0.296–0.947)	0.602 (0.345–0.852)
Multivariate	0.549 (0.229–0.871)	0.976 (0.957–1)	0.932 (0.806–1)	0.525 (0.116–0.947)	0.815 (0.389–1)	0.463 (0.115–0.728)

**Table 3:** LC-MS/MS measurement conditions.

Analyte	Cone [V]	CE [eV]	MRM transition	RT^a^ [min]	Calibration range [μM]	R^2^
Choline	40	15	104.1 > 60.2	2.318 ± 0.011	0.05–49	0.990
Betaine	30	20	118 > 59	1.528 ± 0.007	0.2–49	0.992
Cytidine	35	10	244 > 112	0.769 ± 0.011	0.06–15	0.95
Uridine	35	10	245 > 113	0.671 ± 0.008	0.11–15	0.990
Betaine D_11_	10	25	129 > 66	1.530 ± 0.006	—	—

Note: ^a^ mean (±s) from standards.
